# Editorial: Advancing the measurement, interpretation, and validation of dynamic functional connectivity

**DOI:** 10.3389/fnhum.2023.1206098

**Published:** 2023-05-11

**Authors:** David O'Connor, Catie Chang, Georgios D. Mitsis

**Affiliations:** ^1^Icahn School of Medicine at Mount Sinai, New York, NY, United States; ^2^Departments of Electrical and Computer Engineering, Computer Science, and Biomedical Engineering, Vanderbilt University, Nashville, TN, United States; ^3^Department of Bioengineering, McGill University, Montreal, QC, Canada

**Keywords:** dynamic functional connectivity, functional neuroimaging, validation, statistical modeling, brain states

This special issue attempts to identify novel methodology which bolsters the study of dynamic Functional Connectivity (dFC). There is still much debate as to whether estimates of functional brain relationships over short time scales can be reliably associated with non-imaging phenotypes, and validation of these methods is of paramount importance for the field (Hutchison et al., 2013; Lurie et al., 2020). While human cognitive processes and behavior can vary over short time scales, commonly used methods for estimation of whole brain functional connectivity are limited in terms of their spatial and temporal resolution. Validation of dFC methods can be achieved by brain state manipulation, multimodal imaging/experimentation, more rigorous statistical modeling, or some combination of these approaches. The collection of articles in the current Research Topic present recent advances related to these general topics.

The work by Qin et al. highlights the utility of dFC methods in clinical research and demonstrates the causal effects of a clinical intervention on dFC. In this study, multimodal experimentation methods, in the form of transcranial magnetic stimulation (TMS) and functional magnetic resonance imaging (fMRI), were used as a therapeutic intervention and to assess recovery from stroke. A combination of ICA for brain region definition, sliding windows for dFC calculation, and K Means clustering for state definition, were used to generate metrics for assessing stroke recovery. It was shown that partial recovery of connectivity between sensorimotor and cognitive control domains, as assessed by resting-state fMRI (rsfMRI) before and after the intervention (1- and 3-months post-stroke), was achieved by TMS based rehabilitation. Overall, the use of a causal intervention step suggests that disease recovery, in this case stroke, can potentially be assessed using dFC methods. Lee et al. present another intervention study showing changes in rsfMRI networks induced by cognitive control training. Here, a six-week period of intensive cognitive training resulted in increased integration between functional brain networks, mirroring natural developmental changes in network organization across adolescence. This study further supports the utility of resting-state connectivity measures for assessing functional reconfiguration in brain networks.

An important methodological question is the extent to which dFC measures are reliable across experimental procedures and data acquisition parameters. Cahart et al. investigated the test-retest reliability of single and multiband rsfMRI in healthy older adults using a dynamic analysis technique termed LEiDA. Some prior work has shown that LEiDA is sensitive to cognitive ability changes in an aging cohort (Cabral et al., 2017). The work of Cahart et al. sought to assess the test-retest reliability of this analytical method across different fMRI acquisitions with varying levels of multiband acceleration. Multiband acquisition is highly relevant to dFC studies, as it can reduce the repetition time of fMRI from ~2 s down to below 1 s, potentially enabling dynamic cognitive processes to be more clearly resolved (albeit with complex tradeoffs in terms of signal-to-noise ratio). Thus, studies that investigate the reproducibility of dynamic analysis methods across acquisition parameters can provide valuable information for applying and interpreting the results of dFC.

The investigation of dFC need not be performed at the whole brain level. For instance, a study by Keogh et al. placed deep brain stimulation electrodes in three individuals with medically and surgically refractory neuropathic pain, centralized on the dorsal anterior cingulate cortex (dACC) and investigated short time activation and coherence patterns of the neuronal populations within the dACC. In addition to revealing the characteristics of information flow within the ACC during a decision-making task, their results showed that these network dynamics were altered during learning. This study, along with Wang et al. which recorded local field potentials in mice from mPFC and mediodorsal thalamus, show that dynamic connectivity is altered at the circuit level during cognitive processes. These studies provide a concrete demonstration that specific short time activation and connectivity profiles manifest in response to certain behaviors. How these changes are reflected in more widely applicable macroscopic measurement modalities, however, remains to be determined.

In addition to validation of brain imaging methods, attention must be paid to non-imaging phenotypes being estimated, and how different brain states can impact these measurements. In this context, the work of Brandman et al. is relevant; this study does not use brain imaging, but rather retrospective behavioral sampling to show how brain states can be manipulated over a short time period using naturalistic stimuli. More grounded task paradigms are crucial for shifting findings obtained in a lab setting into the real world in a truly translational manner.

A key challenge in analyzing whole brain dFC is the size of the derived data. This can yield challenges both in terms of computational demands and result interpretation. In work addressing the former issue, Sendi et al. constructed a two-stage dFC pipeline for deriving clusters (representing connectivity states) from large datasets. Crucially, this pipeline was found to be 25 times faster than the conventional procedure of generating connectivity states, while producing states that are highly similar to those of the conventional approach.

Finally, the study of Gonzalez-Castillo et al. addressed dFC interpretability issues, by investigating the use of low-dimensional embedding techniques, which can capture the variance observed in high dimensional methods but render it in a form that is more computationally feasible and more easily interpreted [not yet published].

Collectively, the above studies highlight complementary approaches for relating dynamic measurements of brain connectivity to cognition and behavior, and across spatial and temporal scales. Further, they probe key methodological questions surrounding dFC analyses, such as computational efficiency and reliability across acquisition parameters. Overall, their findings shed new light on factors shaping dynamic brain connectivity and provide directions for future research.

## Author contributions

All authors listed have made a substantial, direct, and intellectual contribution to the work and approved it for publication.
